# Editorial: Protein Misfolding and Spreading Pathology in Neurodegenerative Diseases

**DOI:** 10.3389/fnmol.2019.00312

**Published:** 2020-01-17

**Authors:** Diana F. Lázaro, Arianna Bellucci, Patrik Brundin, Tiago F. Outeiro

**Affiliations:** ^1^Department of Experimental Neurodegeneration, Center for Biostructural Imaging of Neurodegeneration, University Medical Center Gottingen, Göttingen, Germany; ^2^Division of Pharmacology, Department of Molecular and Translational Medicine, University of Brescia, Brescia, Italy; ^3^Center for Neurodegenerative Science, Van Andel Institute, Grand Rapids, MI, United States; ^4^Max Planck Institute for Experimental Medicine, Göttingen, Germany; ^5^The Medical School, Institute of Neuroscience, Newcastle University, Newcastle upon Tyne, United Kingdom

**Keywords:** neurodegenaration, spreading, misfolding proteins, proteinopathy, disease

A common pathological hallmark among different neurodegenerative diseases is the accumulation of aggregated proteins that might cause cellular dysfunction and, eventually, lead to cell death. Amyloid-beta, Tau, alpha-synuclein, TDP-43, or the prion protein, are just a few examples of proteins that can aggregate and contribute to the pathogenesis of neurodegenerative diseases with diverse clinical manifestations (Alzheimer's disease, frontotemporal lobar degeneration, Pick's disease, Parkinson's disease, Lewy body dementia, multiple system atrophy, amyotrophic lateral sclerosis among the most common). Emerging evidence suggests that the progression of symptoms in patients affected by such disorders correlates with the spreading of pathology through the brain, but the molecular mechanisms underlying aggregation and propagation of protein aggregates are still obscure. This Research Topic focuses on the structural and molecular characteristics of aggregation-prone proteins and resumes new aspects of pathology spreading. A series of 10 articles provides an exciting up-to-date overview of core biological features of prion and prion-like neurodegenerative disorders.

Protein aggregation is a common feature among numerous neurodegenerative diseases, and is thought to culminate with detrimental effects for the cells where the proteins accumulate. Despite the commonalities of protein aggregation and cell dysfunction, the pathobiological bases of these diseases may differ. For example, the two characteristic protein deposits in Alzheimer's disease (AD) are the extracellular senile plaques, whose main constituent are amyloid-β (Aβ) fibrils, and intraneuronal neurofibrillary tangles (NFT), composed of hyperphosphorylated Tau protein (Brion et al., 1985, 1986; Kirschner et al., 1986; Sisodia et al., 1990). Similarly, fibrillary alpha-synuclein (aSyn) is the main protein component of Lewy bodies (LB) which are the key neuropathological hallmarks of Parkinson's disease (PD) and Lewy body dementia (DLB) (Spillantini et al., 1997), and can be found within glial cytoplasmic inclusions in the brain of patients affected by multiple system atrophy (MSA) (Wakabayashi et al., 1998). Finally, TAR-DNA binding protein 43 (TDP-43)- or tau-positive inclusions can be found in the brain or spinal cord in, e.g., frontotemporal lobar degeneration (FTLD) or amyotrophic lateral sclerosis (ALS) (Arai et al., 2006; Hasegawa et al., 2008; Kabashi et al., 2008; Sreedharan et al., 2008; Da Cruz and Cleveland, 2011). Recently, Aβ, Tau, aSyn, and TDP-43 were proposed to display several properties similar to those exhibited by the prion protein (PrP). In particular, their physiological conformation can shift to a self-replicating pathological strain which can spread from a neuron to another across different brain regions.

Intriguingly, there seems to be a cross-talk between PD and AD, as aSyn pathology is frequent in AD brains, and Tau accumulation is common in PD with dementia (Lippa et al., 1998; Hamilton, 2000; Irwin et al., 2013). In a similar vein, Aβ plaques are a common feature of DLB, where aSyn aggregates are the predominant feature (McCann et al., 2014).

Remarkably, the prion-like properties of Aβ and aSyn demonstrated in experimental models, have been corroborated by studies that have suggested that human transmission of cerebral amyloid, and Aβ pathology contaminated material, occurs upon certain clinical procedures (Purro et al., 2018; Banerjee et al., 2019; Spinazzi et al., 2019). Furthermore, host to graft propagation of LB pathology has been suggested to have occurred in patients who received fetal neuron transplants over one decade prior to death and neuropathological examination (Kordower et al., 2008a,b; Chu and Kordower, 2010; Li et al., 2010; Kurowska et al., 2011). Whether these clinical observations with Aβ and aSyn pathology represent authentic prion spreading of pathology remains controversial, and this issue calls for caution due to the tremendous implications it might have.

Therefore, using prion-like terminology for all neurodegenerative diseases requires a deeper understanding of the molecular mechanisms involved. There are currently no disease-modifying treatments available for these diseases, and therefore it is desirable to design strategies that could directly target the aggregation of these proteins or modulate their ability to propagate from one brain region to another. Ongoing clinical trials with immunotherapy are focused on clearing the aggregates when they are in the extracellular space, and can be viewed as a strategy to limit prion-like spreading of the pathology (Gallardo and Holtzman, 2017; Sigurdsson, 2018; Panza et al., 2019).

In this Research Topic of Frontiers in Molecular Neuroscience, Terry and Wadsworth review the importance of prion structure for pathogenicity. They highlight and discuss new findings differentiating the architecture of authentic infectious lethal prions from that of PrP amyloidosis as the pillar for a critical re-evaluation of the structure of other prion-like proteins associated with other neurodegenerative diseases (Terry and Wadsworth). In the second paper, Lim reviews the analogy between prion protein and other aggregation-prone proteins, such as aSyn, Aβ, and Tau, focusing on the likelihood of these proteins to cross-seed and to adopt different conformations, and on the importance of understanding the molecular basis that drive the different conformations. Next, Vasili et al. discuss overlapping aspects between PD and AD, and elaborate on the mechanisms involved in the transfer/spreading of aSyn and Tau. They provide a thorough overview of the current cell and animal models to assess the mechanisms of spreading of pathology (Vasili et al.). On the same line, Friesen and Meyer-Luehmann summarize the literature on Aβ seeding in mouse models of AD, and their application for the study of cerebral amyloidosis and associated pathologies.

Prasad et al. present a comprehensive review about the role of TDP-43 in ALS. They discuss the imbalances on TDP-43 homeostasis, implicated in miss-regulation of RNA and cytotoxicity mechanisms. McAlary et al. provide a detailed review of the current evidence on idiopathic ALS as a prion-like disorder. They focus on key proteins and genes involved in the disease (TDP-43, SOD1, FUS, and C9orf72), and discuss the current evidence from biophysical to *in vivo* studies (McAlary et al.).

Chen et al. review the consequences of gut inflammation on PD, highlighting how it may initiate and promote enteric aSyn pathology deposition and spreading into the brain.

The putative contribution of calcium channels to PD etiopathogenesis and progression, putting the accent on how they might contribute to aSyn aggregation and secretion in synucleinopathies, is addressed and discussed in the article by Leandrou et al..

New translational insights on neurodegenerative disorders characterized by prion-like protein spreading are provided by Singh et al., which describe that increased levels of SIRT2 correlate with circulating aSyn in early PD. They propose SIRT2 as potential biomarker for early detection of the disease (Singh et al.). Finally, Nam et al. discuss the efficacy of ALWPs, a combination of oriental herbal medicines with proven efficacy in diabetes mellitus, immune modulation, and owning both neurotrophic and anti-inflammatory action in decreasing Aβ plaque load, as well as Tau hyperphosphorylation in the cortex and hippocampus of the 5XFAD mouse model of AD.

In short, the articles in this Research Topic provide new up-to-date insights into our understanding of protein aggregation and spreading of pathology in prion and prion-like neurodegenerative disorders. Addressing a wide variety of topics, they introduce thought-provoking concepts and clues about the relevance of laboratory findings to the clinical arena. Hopefully, a greater understanding of the prion-like propagation of protein aggregate pathology will lead to novel therapeutic strategies that slow disease progression.

## Author Contributions

All authors listed have made a substantial, direct and intellectual contribution to the work, and approved it for publication.

### Conflict of Interest

The authors declare that the research was conducted in the absence of any commercial or financial relationships that could be construed as a potential conflict of interest.
